# Neutrophil–microglia communication through the C3–C3aR1 axis contributes to secondary neuroinflammation after traumatic brain injury

**DOI:** 10.3389/fimmu.2026.1979527

**Published:** 2026-10-23

**Authors:** Tianrui Ma, Yichi Zhang, Xiran Wang, Xiangru Ren, Linan Jiao, Kaifeng Pang, Chenyang Liu, Zijian Zhou, Pengbo Zhao, Hao Zhang, Ying Fu, Lei Li, Yang Li, Zilong Zhao

**Affiliations:** 1 Department of Neurosurgery, Tianjin Neurological Institute, State Key Laboratory of Experimental Hematology, Laboratory of Post-Neuroinjury Neurorepair and Regeneration in Central Nervous System Tianjin and Ministry of Education, Tianjin Medical University General Hospital, Tianjin, China; 2 Department of Physical and Rehabilitation Medicine, Tianjin Medical University General Hospital, Tianjin, China; 3 Department of Neurology and Institute of Neurology of First Affiliated Hospital, Institute of Neuroscience, and Fujian Key Laboratory of Molecular Neurology, Fujian Medical University, Fuzhou, China; 4 Department of Neurosurgery, Tianjin HuanHu Hospital, Tianjin, China; 5 Precision Medicine Center, Tianjin Medical University General Hospital, Tianjin, China

**Keywords:** C3aR1, complement C3, microglia, neuroinflammation, neutrophils, traumatic brain injury

## Abstract

**Background:**

Traumatic brain injury (TBI) triggers a rapidly evolving neuroinflammatory response that contributes to secondary brain injury. Although infiltrating leukocytes and resident microglia are both prominent components of this response, the cellular signals that connect peripheral immune-cell recruitment to microglial state remodeling remain incompletely defined.

**Methods:**

We integrated and reanalyzed publicly available single-cell RNA-sequencing datasets from mouse TBI models to define temporal changes in brain-cell composition and transcriptional states. CellChat and NicheNet were used to prioritize candidate neutrophil-to-microglia signaling pathways. The predicted cellular distribution of C3 and C3aR1 was evaluated by immunofluorescence and flow cytometry. The effects of JR14a treatment on microglial inflammatory responses, blood–brain barrier (BBB) integrity, and neurological outcomes were examined in a mouse TBI model. Single-Cell rEgulatory Network Inference and Clustering (SCENIC), CellOracle, a gene regulatory network inference and in silico transcription-factor perturbation framework, and scTenifoldKnk, a single-cell gene regulatory network-based virtual knockout framework, were used for gene regulatory network inference and in silico transcription-factor perturbation analyses in *C3ar1*-high microglia.

**Results:**

Analysis of 254,512 cells revealed a transient increase in infiltrating neutrophils during the acute phase of TBI and the emergence of inflammatory microglial states. CellChat and NicheNet independently prioritized C3-associated signaling between neutrophils and Microglia4. C3 expression was enriched in infiltrating neutrophils, whereas *C3ar1* was preferentially expressed by inflammatory microglia. Flow cytometry showed that C3aR1-positive microglia expressed higher levels of IL-1β and TNF-α than C3aR1-negative microglia. JR14a treatment reduced microglial inflammatory cytokine production, preserved Claudin-5 expression, limited Evans blue extravasation, and improved neurological and cognitive outcomes after TBI. Exploratory regulatory analyses further prioritized Ets2, Fosl2, and Irf7 as candidate regulators of the *C3ar1*-high microglial program.

**Conclusions:**

These findings implicate a neutrophil-associated C3/C3a–C3aR1 communication pathway in inflammatory microglial remodeling after TBI and support further investigation of C3aR1-associated signaling as a therapeutic target for secondary neuroinflammation.

## 1 Introduction

Traumatic brain injury (TBI) is a major cause of death and long-term neurological disability worldwide (1, 2). The initial mechanical insult is followed by secondary processes that include neuroinflammation, oxidative stress, blood–brain barrier (BBB) disruption, and neuronal dysfunction. These responses evolve over hours to days and substantially influence tissue loss and neurological recovery (3–5). Despite advances in critical care, treatments that specifically limit secondary injury remain limited, in part because the cellular interactions that sustain post-traumatic inflammation are not fully understood.

Microglia, the resident immune cells of the central nervous system (CNS), play essential roles in maintaining tissue homeostasis and responding to brain injury (6–8). Following TBI, microglia rapidly undergo profound transcriptional and functional changes, adopting diverse activation states ranging from reparative to highly inflammatory phenotypes. Some responses support debris clearance and tissue repair, whereas persistent inflammatory activation can amplify neuronal and vascular injury through cytokines, chemokines, and complement-associated pathways (9, 10). Recent single-cell transcriptomic studies have revealed substantial heterogeneity among microglial populations after CNS injury, indicating that microglial activation is shaped by diverse local and systemic signals. These include damage-associated molecules, inflammatory cytokines and chemokines, complement factors, oxidative stress, and mediators released by infiltrating immune cells after blood–brain barrier disruption (11–13). However, the upstream cellular signals responsible for directing microglial state transitions after TBI remain incompletely understood.

Peripheral immune cell infiltration is a prominent feature of acute TBI and contributes to the inflammatory microenvironment within the injured brain (14, 15). Among infiltrating immune populations, neutrophils are among the earliest recruited cells and accumulate rapidly within hours to days after injury (16–18). Activated neutrophils can influence tissue injury through the release of reactive oxygen species, proteases, extracellular traps, and inflammatory mediators (19–21). Although neutrophils have traditionally been considered direct effectors of tissue damage, increasing evidence suggests that they also function as regulatory immune cells capable of shaping responses of resident CNS cells through cytokine and ligand–receptor signaling (22, 23). However, whether infiltrating neutrophils actively regulate microglial phenotype transition after TBI and the molecular mechanisms involved remain poorly defined.

Here, we integrated public single-cell transcriptomic datasets with cell–cell communication analyses, histological and flow-cytometric validation, and pharmacological intervention to examine neutrophil–microglia communication after TBI. We identified a putative C3/C3a–C3aR1 axis linking infiltrating neutrophils to an inflammatory microglial state. We further evaluated the association of this pathway with microglial cytokine production, BBB disruption, and neurological outcomes and used complementary computational approaches to prioritize transcriptional regulators of *C3ar1*-high microglia.

## 2 Results

### 2.1 Single-cell transcriptomic profiling reveals dynamic immune remodeling after traumatic brain injury

To characterize the cellular landscape following traumatic brain injury (TBI), we integrated and analyzed single-cell RNA-sequencing of mouse brains collected from control (n=10), 1 day post-injury (TBI1d, n=9), and 7 days post-injury (TBI7d, n=3). Following quality control and data integration, 254,512 high-quality cells were retained for subsequent analyses (**Figure 1A**). Unsupervised clustering, validated by canonical marker gene expression (**Figure 1B**), identified major brain and immune cell populations, including microglia, monocytes/macrophages, neutrophils, endothelial cells, astrocytes, neurons, ependymal cells, pericytes, and choroid plexus cells (**Figure 1A**). TBI induced marked changes in the cellular composition of the injured brain. Monocytes/macrophages and neutrophils increased prominently at TBI1d, whereas the neutrophil fraction declined by TBI7d (**Figure 1C**). Differential-expression analysis showed broad transcriptional remodeling across multiple cell types at both time points (**Figure 1D**; **Supplementary Figures S1A, B**). At TBI1d, microglia, monocytes/macrophages, and neutrophils were enriched for programs related to leukocyte migration, cell adhesion, inflammatory regulation, and apoptotic signaling, with cell type-specific differences in the dominant pathways (**Figure 1E**). By TBI7d, enrichment shifted toward RNA processing, hematopoietic regulation, cellular respiration, and oxidative phosphorylation (**Figure 1F**). Direct comparison between TBI1d and TBI7d further demonstrated this temporal transition. Innate immune populations at TBI1d were more strongly associated with leukocyte migration and apoptotic signaling, whereas cells at TBI7d showed greater enrichment of energy metabolism, cellular respiration, and neural remodeling-associated processes (**Supplementary Figures S1C, D**). These findings indicate that TBI is accompanied by dynamic, cell type–specific immune remodeling, with an acute inflammatory phase followed by broader metabolic and biosynthetic reprogramming.

**Figure 1:**
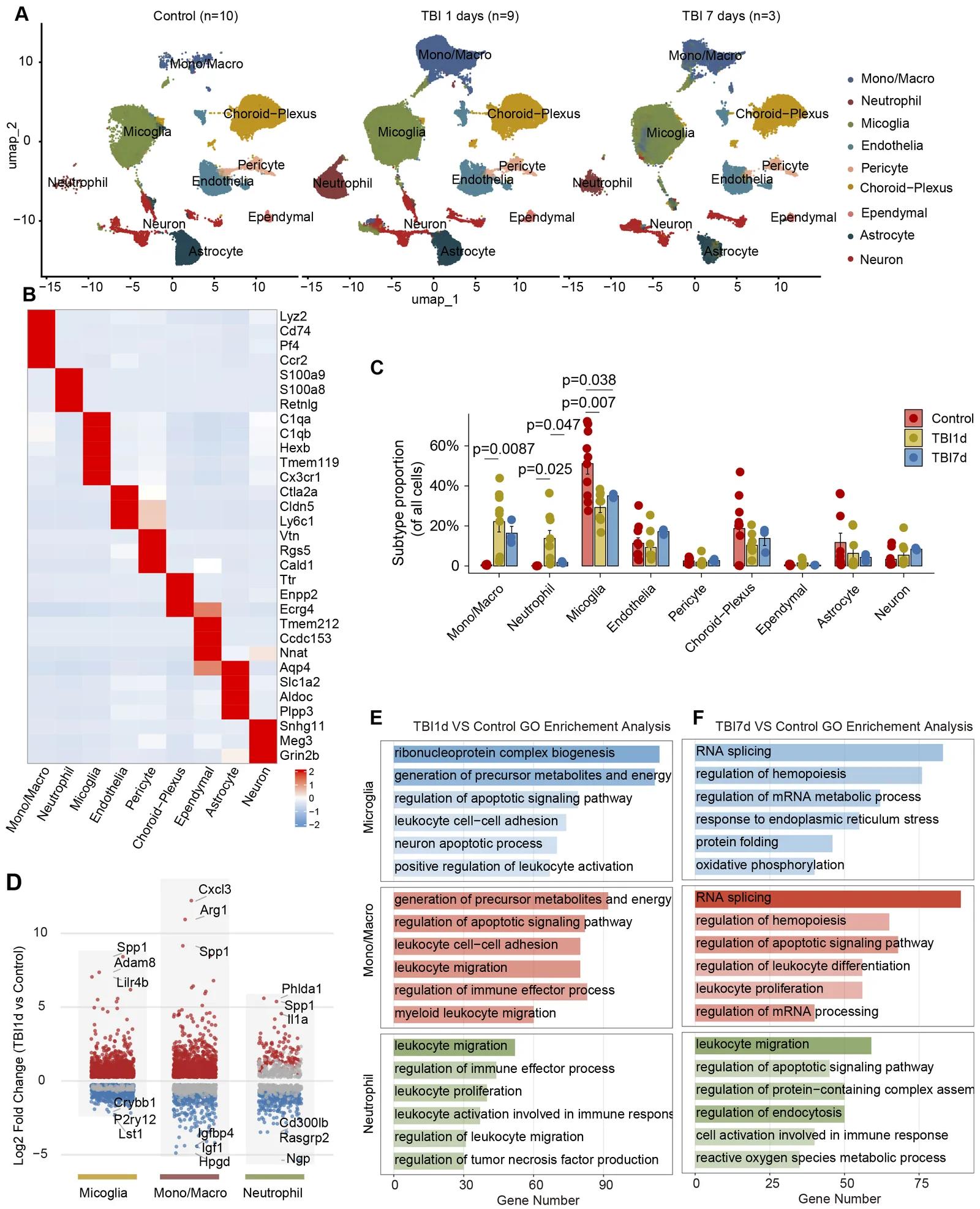
Single-cell transcriptomic landscape revealed dynamic cellular remodeling after TBI. **(A)** Uniform Manifold Approximation and Projection (UMAP) visualization of integrated single-cell RNA sequencing data from Control, TBI1d, and TBI7d groups, showing the distribution of major brain cell populations, including monocytes/macrophages, neutrophils, microglia, endothelial cells, pericytes, choroid plexus cells, ependymal cells, astrocytes, and neurons. **(B)** Heatmap displaying the representative marker genes used for cell-type annotation. **(C)** Quantification of the relative proportions of each major cell population across Control, TBI1d, and TBI7d groups, demonstrating dynamic changes in immune and resident brain cell composition following TBI (one-way ANOVA). **(D)** Plot showing differentially expressed genes (DEGs) in neutrophils, mono/macro and microglia between the TBI1d and Control groups. Red dots indicate significantly upregulated genes (P < 0.01, log2FC ≥ 0.26), blue dots indicate significantly downregulated genes (P < 0.01, log2FC ≤ −0.26), and gray dots represent genes without significant differential expression. **(E, F)** Gene Ontology (GO) enrichment analysis of differentially expressed genes in monocytes/macrophages, microglia, and neutrophils comparing each TBI group with the Control group [**(E)**, TBI1d vs. Control; **(F)**, TBI7d vs. Control]. Color opacity represents −log10(adjusted *P* value), and all displayed pathways had adjusted *P* values < 0.05. Statistical analysis was performed by one-way ANOVA with Tukey *post hoc* tests **(C)**.

### 2.2 TBI induces heterogeneous microglial state remodeling

Given that microglia represent the principal resident immune cells in the central nervous system, we next investigated their heterogeneity following TBI. Reclustering analysis identified 7 transcriptionally distinct microglial subpopulations, each exhibiting unique molecular signatures (**Figures 2A–C**). The relative abundance of these subsets shifted substantially post-injury, indicating a dynamic remodeling of the microglial states. Notably, Microglia3 and Microglia4 underwent marked expansion at TBI1d, followed by a decline in their relative abundance at TBI7d (**Figure 2B**). Functional annotation further revealed that Microglia1 and Microglia2 were associated with RNA processing, leukocyte differentiation, and negative regulation of immune-cell activation; Microglia3 was enriched for ribonucleoprotein-complex and ribosome biogenesis; and Microglia4–7 showed prominent enrichment of metabolic programs, including oxidative phosphorylation, cellular respiration, and ATP synthesis (**Supplementary Figures S2A–G**). To further characterize the functional states of these microglial subpopulations, we calculated module scores for inflammatory response, phagocytosis, and lipid metabolism using gene sets listed in **Supplementary Tables S1**-**S3**, respectively. Notably, the TBI1d-enriched Microglia3 and Microglia4 exhibited significantly elevated inflammatory scores compared with the other subsets, whereas microglia at TBI7d displayed significantly increased lipid metabolism scores (**Figures 2D–F**). These distinct transcriptomic and functional profiles collectively suggest that TBI drives the emergence of inflammatory microglia. To further delineate the temporal dynamics of these microglial state transitions, we performed pseudotime trajectory analysis using Monocle2. Microglia distributed along a continuous transcriptional trajectory from homeostatic states (Microglia2) toward activated states characterized by inflammatory and metabolic remodeling, particularly Microglia3 and Microglia4 (**Figures 2G, H**). To further characterize transcriptional divergence at a defined region of the microglial trajectory, we performed Branched Expression Analysis Modeling (BEAM) focusing on branch point 5 of the Monocle2 trajectory. BEAM identified 590 genes whose expression dynamics differed significantly between the alternative cell-state trajectories emerging from this branch point and separated these genes into four expression modules (**Figure 2I**). GO enrichment analysis linked these modules to biological processes including leukocyte migration, positive regulation of inflammatory responses, neuronal apoptotic processes, RNA processing, ATP metabolism, lipid biosynthesis, and glial cell development (**Figure 2J**). Thus, transcriptional divergence at branch point 5 was accompanied by coordinated changes in inflammatory, biosynthetic, and metabolic programs. Collectively, these findings underscore that TBI induces profound microglial state transitions that are tightly coupled with acute inflammatory activation.

**Figure 2:**
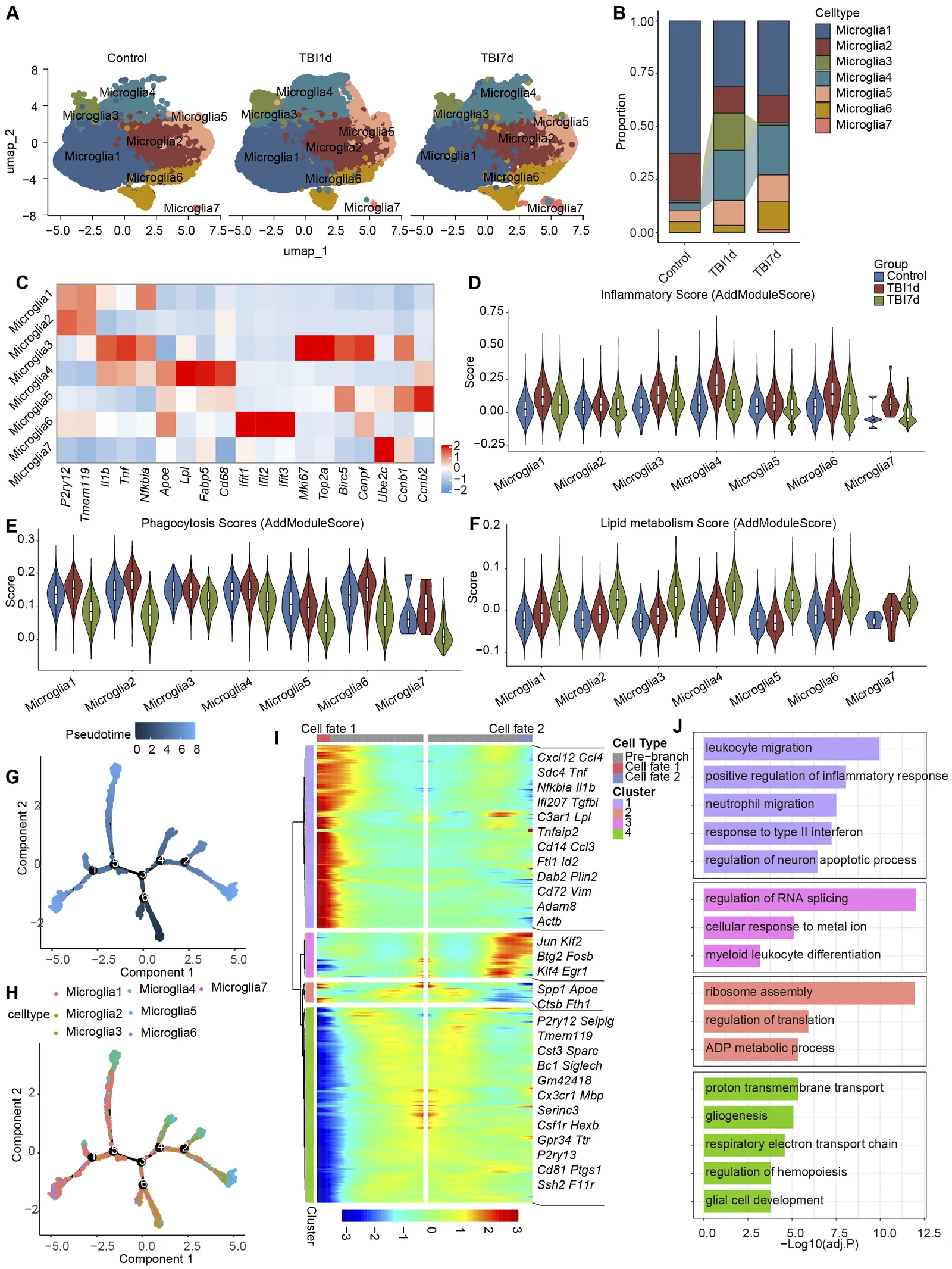
Single-cell analysis revealed dynamic inflammatory and metabolic remodeling of microglia following TBI. **(A)** UMAP visualization showing 7 transcriptionally distinct microglial subclusters in the Control, TBI1d, and TBI7d groups. **(B)** Relative abundance of each microglial subcluster across Control, TBI1d, and TBI7d groups. **(C)** Heatmap displaying representative marker genes for each microglial subcluster. Color intensity indicates the scaled average expression level of each marker gene. **(D–F)** Comparison of inflammatory scores **(D)**, phagocytosis scores **(E)** and lipid metabolism scores **(F)** among different microglial subclusters in the Control, TBI1d, and TBI7d groups using AddModuleScore analysis. The gene sets used for these analyses are provided in **Supplementary Tables S1**-**S3**, respectively. **(G)** Monocle pseudotime trajectory analysis depicting the dynamic transcriptional transition of microglial cells during disease progression. **(H)** Pseudotime trajectory colored according to microglial subclusters, illustrating the distribution of each subpopulation along the inferred transcriptional state-transition trajectory. **(I)** Heatmap showing branch-dependent gene expression dynamics identified by Branched Expression Analysis Modeling at branch point 5 of the Monocle2 trajectory. Genes were clustered into four modules according to their differential expression patterns along the two downstream microglial state-transition paths. **(J)** GO enrichment analysis of branch-dependent genes identified by BEAM.

### 2.3 Cell–cell communication analysis identifies neutrophil-associated C3 signaling targeting activated microglial states

Neutrophils accumulated prominently at TBI1d and displayed transcriptional programs related to inflammatory responses and cytokine production (**Figures 1C–E**). This temporal overlap between neutrophil recruitment and the expansion of activated microglia states prompted us to investigate potential signaling interactions between these populations. CellChat analysis showed increased outgoing communication from neutrophils to microglial populations at TBI1d, with prominent predicted interactions involving Microglia2, Microglia3, and Microglia4 (**Figures 3A, B**). Comparison across time points further identified several ligand–receptor pairs enriched during the acute phase, including THBS1–SDC4, CCL3/CCL4–CCR5, and C3–C3aR1 (**Figure 3C**). We next used NicheNet, with neutrophils defined as sender cells and Microglia4 as receiver cells, to prioritize ligands associated with the receiver-cell transcriptional program. Cd9, Lgals3, and C3 ranked among the leading candidate ligands (**Figure 3D**). Among the candidate pathways, C3/C3a–C3aR1 was prioritized because it was independently identified by both CellChat and NicheNet, showed complementary cell-type-specific ligand–receptor expression, and has established biological relevance to neuroinflammation after TBI. C3 transcript expression was most prominently enriched in infiltrating neutrophils at TBI1d, whereas *C3ar1* was enriched in Microglia4 and varied along the inferred microglial trajectory (**Figures 3E, F**). Predicted C3-responsive genes were enriched for leukocyte activation, inflammatory responses, cytokine production, and innate immune signaling (**Figure 3G**). Consistently, *C3ar1*-high microglia showed stronger inflammatory and immune-related transcriptional programs than *C3ar1*-low microglia (**Figures 3H, I**). Together, these analyses nominate neutrophil-associated C3 as a candidate signal linked to the inflammatory *C3ar1*-high microglial state.

**Figure 3:**
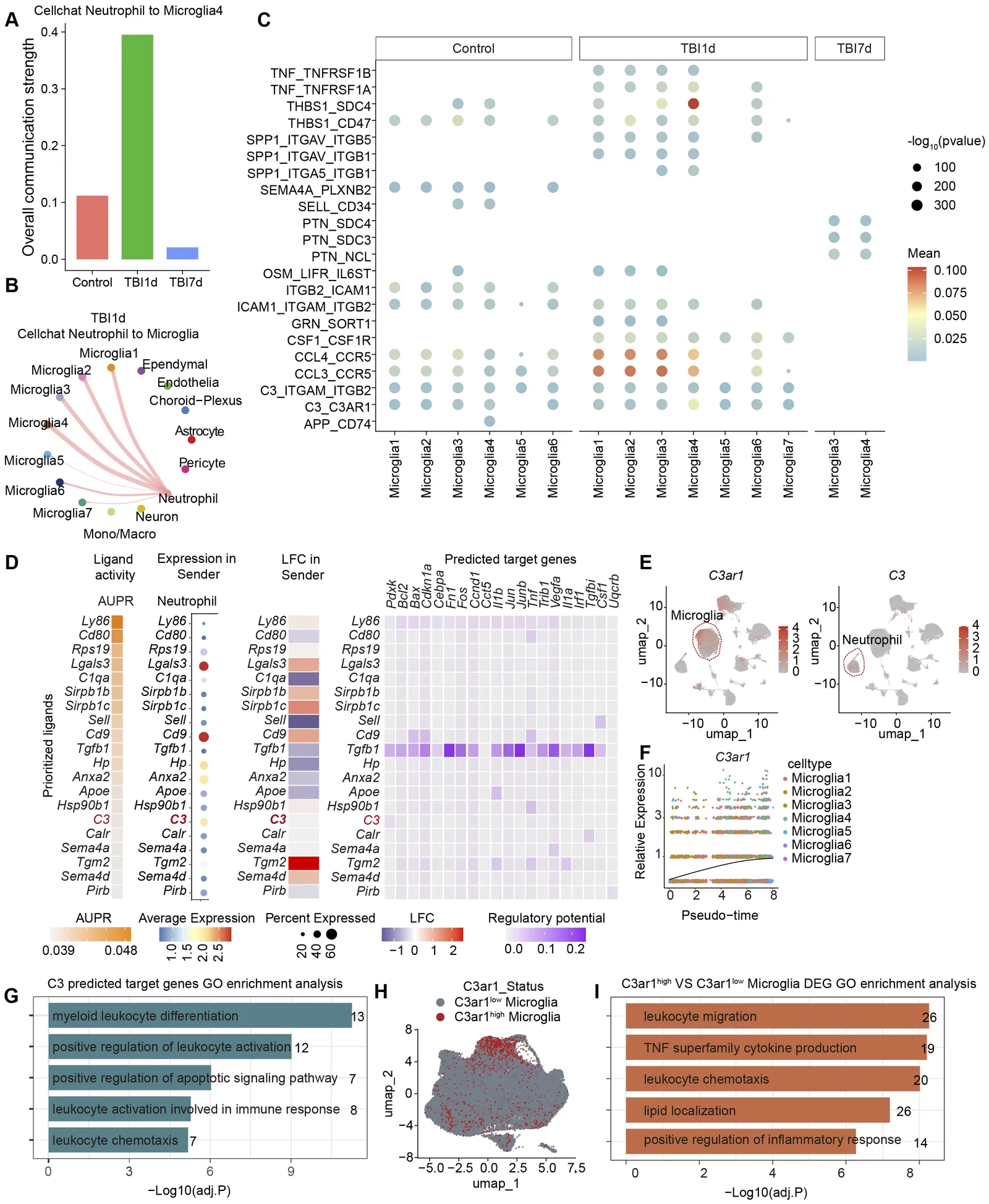
Cell–cell communication analysis identified the neutrophil-associated C3/C3a–C3aR1 signaling axis as a potential regulator of inflammatory microglial activation following TBI. **(A)** Overall interaction strength between neutrophils and Microglia4 in the Control, TBI1d, and TBI7d groups. **(B)** Circle plot showing the inferred communication network from neutrophils to microglial subpopulations at TBI1d. **(C)** Bubble plot showing ligand–receptor interactions between neutrophils and individual microglial subsets in the Control, TBI1d, and TBI7d groups. Dot size represents the statistical significance of each interaction (−log_10_ *P* value), and color indicates the communication probability. **(D)** NicheNet analysis identifying candidate neutrophil-derived ligands regulating Microglia4. The left panels show ligand activity, quantified by the area under the precision–recall curve (AUPR), ligand expression in neutrophils, and differential ligand expression between experimental groups. AUPR reflects the ability of each ligand–target model to recover the receiver-cell gene program, with higher values indicating greater predicted ligand activity. The right panel illustrates the predicted ligand–target regulatory network. **(E)** Feature plots showing the expression of C3ar1 in microglia and C3 in neutrophils. **(F)** Dynamic expression of C3ar1 across the microglial pseudotime trajectory, illustrating its transcriptional changes during microglial state transition. **(G)** GO-BP enrichment analysis of all C3 target genes predicted by NicheNet. Enrichment analysis was performed using clusterProfiler, and GO terms with Benjamini–Hochberg-adjusted P values < 0.05 were considered significant. **(H)** UMAP visualization showing the distribution of *C3ar1*-high and *C3ar1*-low microglia. **(I)** GO enrichment analysis of differentially expressed genes between *C3ar1*-high and *C3ar1*-low microglia.

To further explore transcriptional regulatory programs associated with the *C3ar1*-high microglial state, we applied three complementary computational approaches: Single-Cell rEgulatory Network Inference and Clustering (SCENIC) for regulon inference, CellOracle for gene regulatory network inference and in silico transcription-factor perturbation, and scTenifoldKnk for single-cell gene regulatory network-based virtual knockout analysis. SCENIC identified *Ets2, Fosl2, Irf7, Mxd1*, and *Tfdp1* among the regulons with high specificity in *C3ar1*-high microglia (**Figure 4A**). Pathway enrichment and comparison of predicted target sets with differentially expressed genes further prioritized *Irf7*, *Ets2*, and *Fosl2*, with the *Irf7* target set showing the greatest overlap with genes differentially expressed in *C3ar1*-high microglia (**Figures 4B, C**). CellOracle simulations predicted that perturbation of these factors could alter transcriptional flow within this microglial population (**Figures 4D–J**). scTenifoldKnk virtual knockout further predicted distinct but partially overlapping perturbation-sensitive gene networks associated with *Ets2*, *Fosl2*, and *Irf7* (**Supplementary Figures S3A–C**). These networks included genes related to inflammatory and stress responses, microglial activation, and metabolic regulation, with functional enrichment implicating inflammatory signaling, lipid metabolism, oxidative stress, and ERK-related pathways (**Supplementary Figures S3D–F**). Together, these hypothesis-generating analyses prioritize *Ets2*, *Fosl2*, and *Irf7* as candidate regulators associated with the *C3ar1-high* microglial state for future experimental validation.

**Figure 4:**
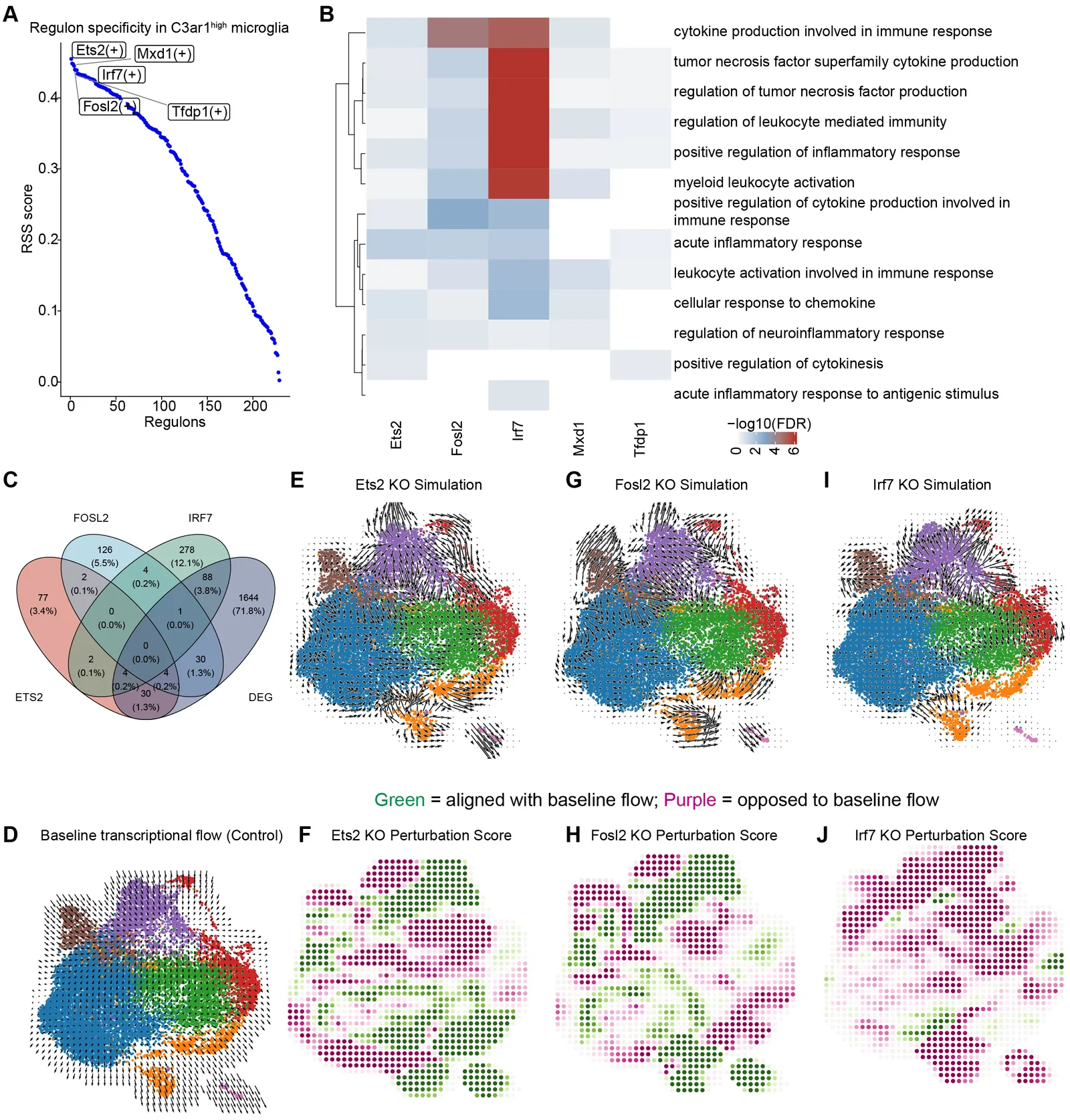
Integrative computational analyses prioritize candidate transcriptional regulators associated with C3ar1-high microglia. **(A)** SCENIC analysis showing regulon specificity scores (RSS) of transcription factors in *C3ar1*-high microglia. *Ets2, Fosl2, Irf7, Mxd1*, and *Tfdp1* ranked among the top regulons with high specificity. **(B)** GO-BP enrichment analysis of predicted target gene sets associated with the selected transcription factors; terms with Benjamini–Hochberg-adjusted P values < 0.05 were considered significantly enriched. **(C)** Venn diagram illustrating the overlap between DEGs in *C3ar1*-high microglia and the predicted target genes of Ets2, Fosl2, and Irf7. **(D)** Baseline transcriptional vector field inferred by CellOracle before in silico perturbation. **(E, F)** CellOracle simulation of *Ets2* perturbation **(E)** and the corresponding perturbation score **(F)**. **(G, H)** CellOracle simulation of *Fosl2* perturbation **(G)** and the corresponding perturbation score **(H)**. **(I, J)** CellOracle simulation of *Irf7* perturbation **(I)** and the corresponding perturbation score **(J)**. Positive perturbation scores indicate alignment of the simulated perturbation with the inferred state-transition direction, whereas negative scores indicate opposition to that transition.

### 2.4 JR14a treatment attenuates microglial inflammatory activation and preserves BBB integrity

We next examined whether the predicted cellular distribution of C3 and C3aR1 could be detected in the injured brain. Immunofluorescence showed increased C3aR1 immunoreactivity in IBA1-positive microglia after TBI (**Figures 5A, B**). Meanwhile, C3 expression was detected in infiltrating neutrophils, indicating neutrophils may represent a potential source of C3 during acute brain injury (**Figures 5C, D**). Single-cell analysis further showed that neutrophil *C3* expression was most prominent at TBI1d and markedly decreased by TBI7d (**Supplementary Figures S5A, B**). Consistent with this temporal pattern, C3-expressing Ly6G^+^ neutrophils remained detectable at TBI7d, but at a relatively low frequency (**Supplementary Figure S5C**). Flow cytometry confirmed an increase in CD45^int^CD11b^+^C3aR1^+^ microglia after TBI (**Figures 5E, F**; **Supplementary Figure S4A**). Within the microglial compartment, C3aR1^+^ microglia showed higher IL-1β and TNF-α expression than C3aR1^-^ microglia (**Figure 5G**). Together, these findings support the association of C3aR1 expression with an inflammatory microglial phenotype after TBI.

**Figure 5:**
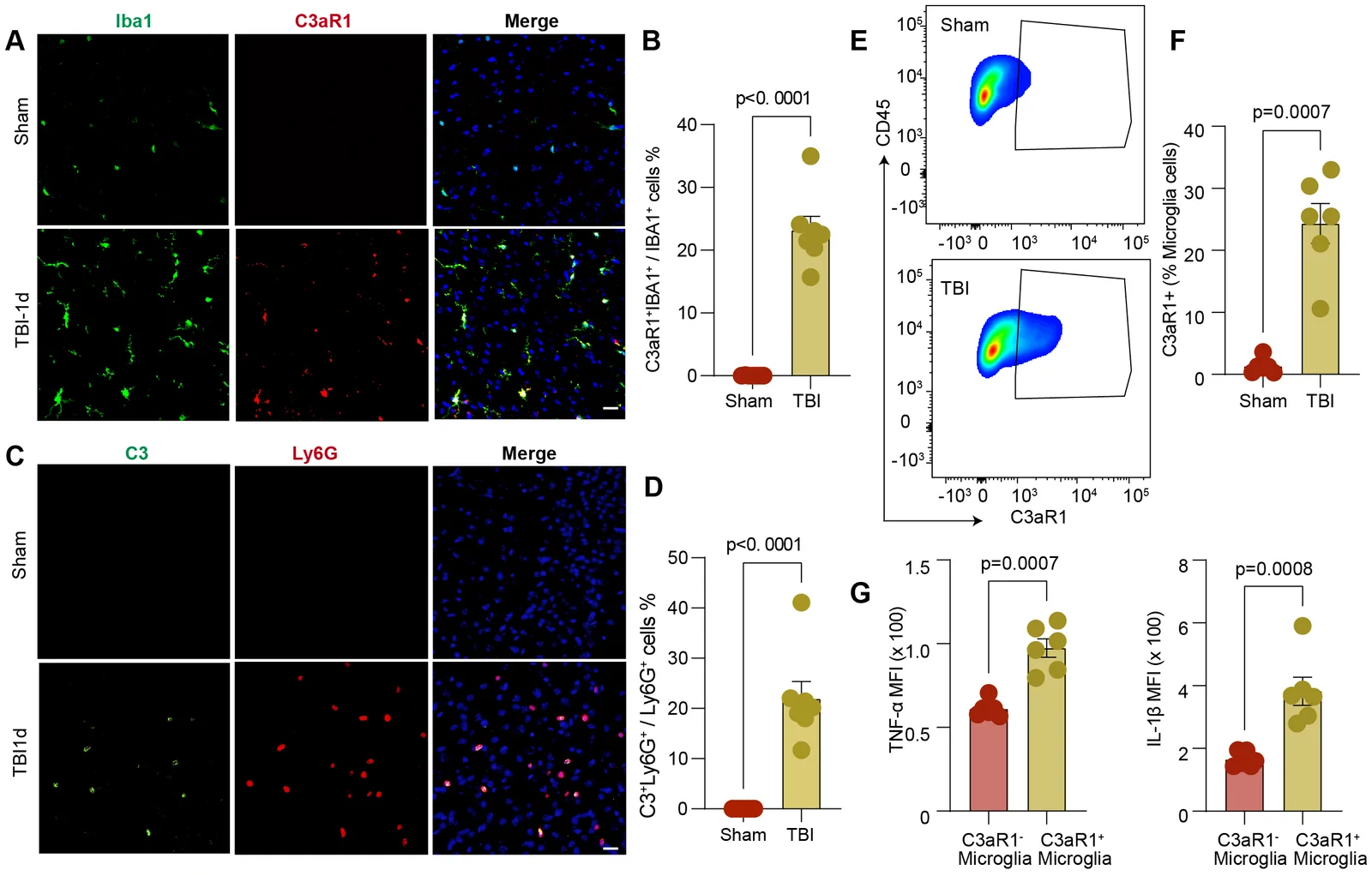
TBI induced neutrophil-associated C3 accumulation and microglia C3aR1 expression upregulation in mice. **(A, B)** Representative immunofluorescence images **(A)** and quantification **(B)** showing co-localization of IBA1 and C3aR1 in the brain of Sham and TBI mice (n=7/group, bar = 20 μm, unpaired t test). **(C, D)** Representative immunofluorescence images **(C)** and quantification **(D)** showing co-localization of C3 and Ly6G in brain from Sham and TBI mice (n=7/group, bar = 20 μm, unpaired t test). **(E)** Representative flow-cytometry plots showing C3aR1 expression within the pre-gated CD45^int^CD11b^+^ microglial population. **(F)** Flow cytometric quantification of C3aR1^+^ microglia in Sham and TBI mice (n=6/group, unpaired t test). **(G)** Flow cytometric analysis of inflammatory cytokine expression in C3aR1^+^ and C3aR1^-^ microglia (n=6/group, paired t test). Data are presented as mean ± SEM.

To determine whether pharmacological modulation of this pathway altered the acute inflammatory response, TBI mice were treated with JR14a (**Figure 6A**). JR14a administration reduced IL-1β and TNF-α expression in C3aR1^+^ microglia (**Figures 6B, C**). Because excessive inflammation contributes to BBB disruption after TBI, we examined vascular integrity. Immunofluorescence staining showed that TBI caused substantial loss of endothelial tight junction protein Claudin-5, whereas JR14a treatment preserved Claudin-5 expression (**Figures 6D, E**). Evans blue leakage analysis further showed reduced BBB permeability following JR14a treatment (**Figures 6F, G**). Together, these findings indicate that JR14a treatment is associated with attenuated microglial inflammatory activation and preservation of BBB integrity after TBI.

**Figure 6:**
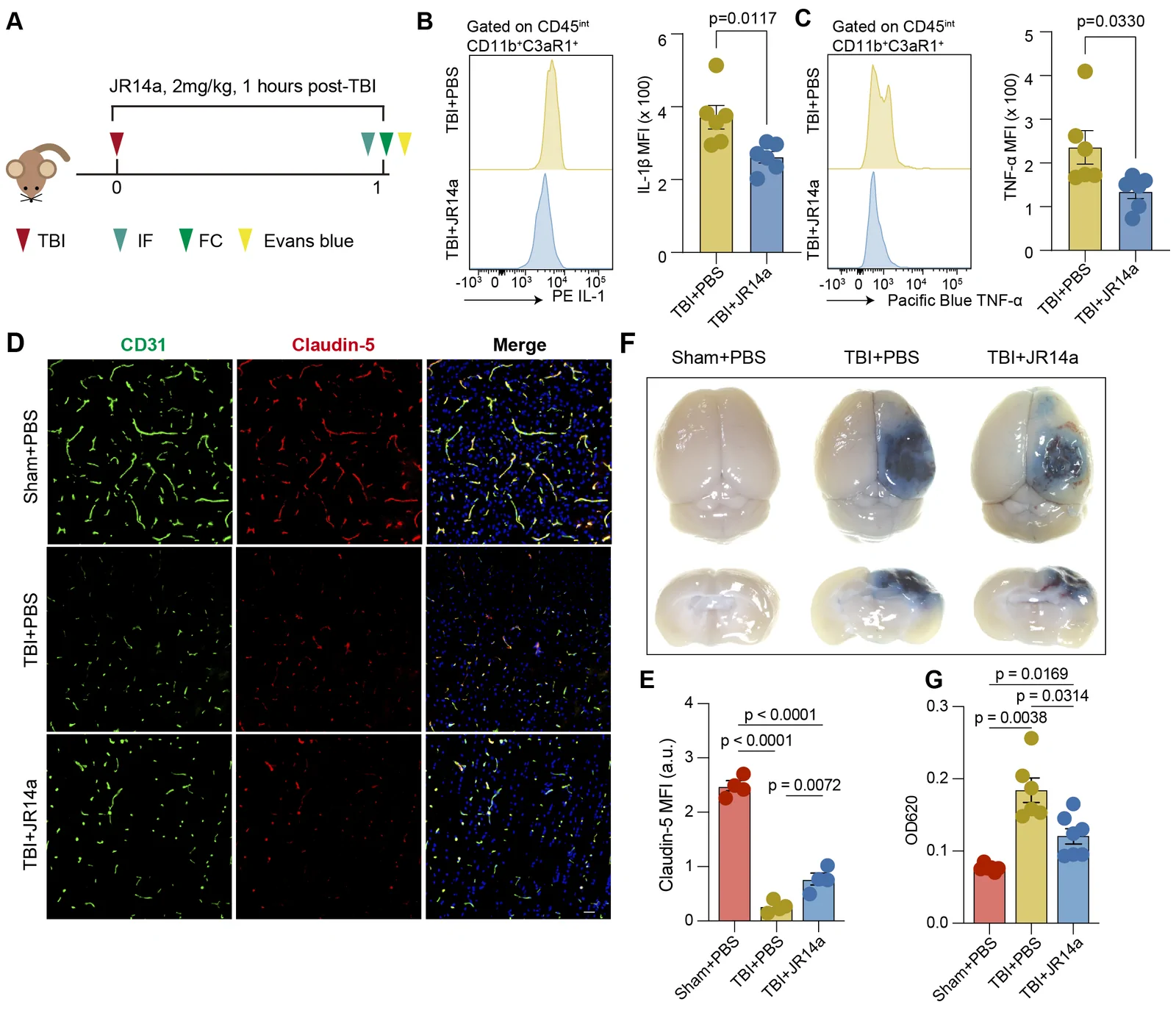
JR14a treatment attenuated microglial inflammatory activation and BBB disruption after TBI. **(A)** Experimental design for the acute JR14a-treatment cohort. Mice received a single dose of JR14a (2 mg/kg, tail vein) within 1 h after TBI and were euthanized 24 h later for flow cytometry, immunofluorescence, and Evans blue permeability assessment. **(B, C)** Representative flow cytometry histograms and quantification showing IL-1β **(B)** and TNF-α **(C)** expression in C3aR1^+^ microglia from TBI, and JR14a-treated TBI mice (n=6/group, unpaired t test). **(D, E)** Representative immunofluorescence images **(D)** and quantification **(E)** of Claudin-5 fluorescence intensity, demonstrating preservation of tight junction protein expression following JR14a treatment (bar = 20 μm, n=7/group, one-way ANOVA test). **(F)** Representative images of Evans blue leakage in brain tissue showing increased vascular permeability after TBI and attenuation following JR14a treatment. **(G)** Quantification of Evans blue extravasation in Sham, TBI, and JR14a-treated TBI mice (n=7/group, one-way ANOVA test). Data are presented as mean ± SEM. Statistical analysis was performed by one-way ANOVA with Tukey *post hoc* tests **(E, G)**.

### 2.5 JR14a treatment improves neurological and cognitive outcomes after TBI

We next asked whether the histological and inflammatory changes associated with JR14a treatment were accompanied by functional improvement (**Figure 7A**). Compared with Sham mice, TBI mice exhibited significant neurological deficits, reflected by increased modified neurological severity scores (mNSS) and impaired motor coordination in the rotarod test. JR14a treatment significantly improved neurological performance during the acute recovery phase (**Figures 7B, C**). To evaluate cognitive function, mice underwent Morris water maze (MWM) testing during the late recovery period. TBI mice showed prolonged escape latency and impaired spatial learning, whereas JR14a-treated mice exhibited improved learning performance (**Figures 7D, E**). During the probe trial, JR14a treatment increased platform crossings without affecting swimming speed, indicating improved spatial memory rather than altered motor capacity (**Figures 7F, G**). Together, these results indicate that JR14a treatment improves acute neurological recovery after TBI.

**Figure 7:**
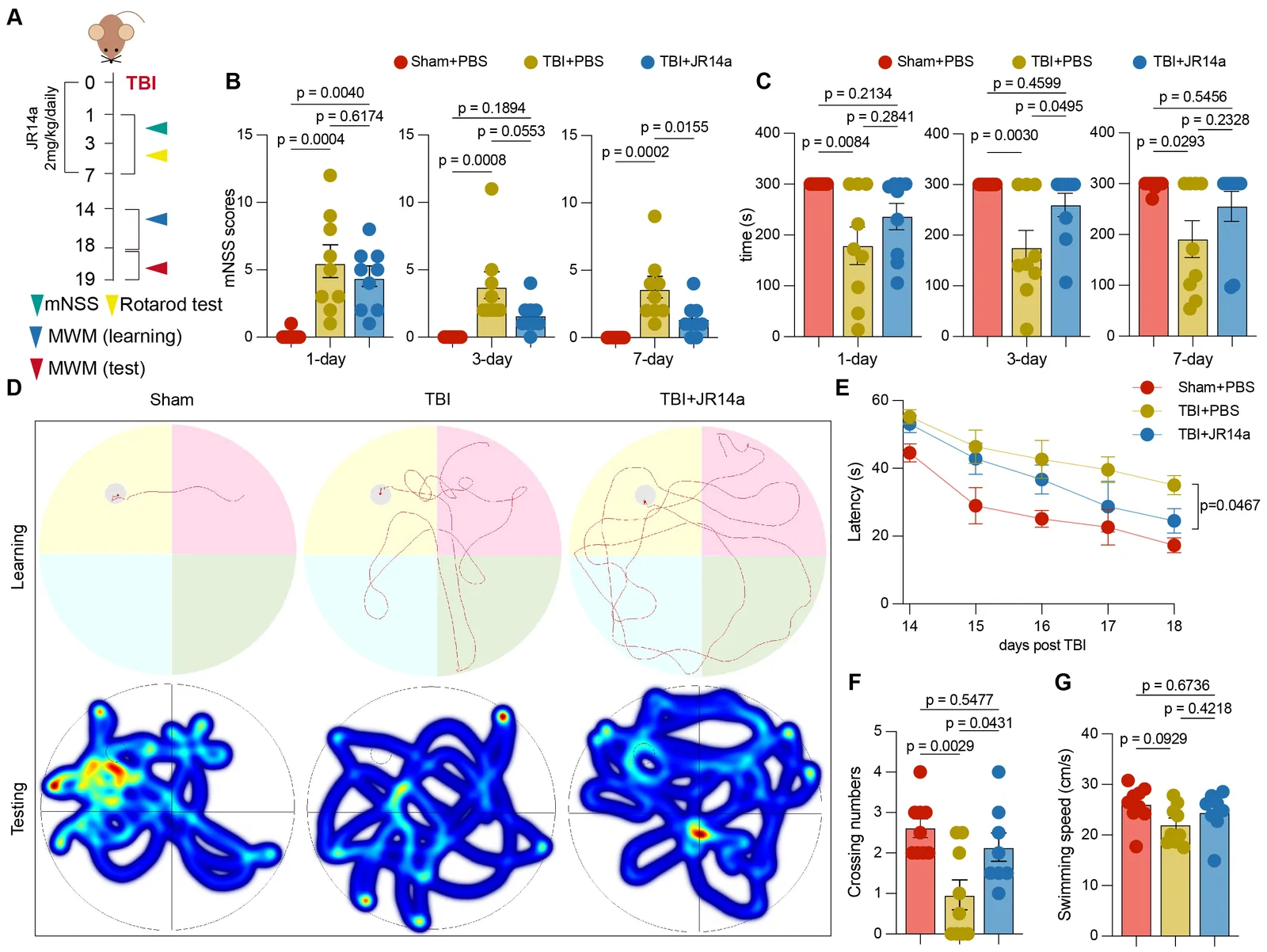
JR14a treatment improved neurological and cognitive outcomes after TBI. **(A)** Experimental timeline illustrating the administration of JR14a and the schedule of behavioral assessments following TBI. **(B)** Modified Neurological Severity Score (mNSS) was assessed at 1, 3, and 7 days after TBI (n=9/group, one-way ANOVA test). **(C)** Rotarod test showing the latency to fall at 1, 3, and 7 days after TBI (n=9/group, one-way ANOVA test). **(D)** Representative swimming trajectories during the learning/acquisition phase (upper panels) and occupancy heat maps during the probe test (lower panels) in Sham, TBI, and JR14a-treated TBI mice. **(E)** Escape latency during Morris water maze (MWM) training from 14 to 18 days after TBI (n=8-9/group, two-way ANOVA test). **(F)** Quantification of platform crossing numbers during the probe trial (n=8-9/group, one-way ANOVA test). **(G)** Average swimming speed during the probe trial, showing no significant difference among groups (n=8-9/group, one-way ANOVA test). Data are presented as mean ± SEM. Statistical analysis was performed by one-way ANOVA with Tukey *post hoc* tests **(B, C, F, G)**.

## 3 Discussion

This study integrates single-cell transcriptomics, computational communication analysis, cellular validation, and pharmacological intervention to examine how infiltrating neutrophils may influence microglial responses after TBI. Four findings emerge from the analysis. First, acute TBI was characterized by transient neutrophil accumulation and expansion of activated microglial states with inflammatory and metabolic features. Second, CellChat and NicheNet independently prioritized C3-associated signaling between neutrophils and Microglia4. Third, *C3ar1*-high microglia displayed enhanced inflammatory transcriptional programs, whereas flow cytometry showed increased IL-1β and TNF-α expression in C3aR1^+^ microglia, and JR14a treatment was associated with reduced microglial activation and preserved BBB integrity. Finally, exploratory network analyses nominated Ets2, Fosl2, and Irf7 as candidate regulators of the *C3ar1*-high microglial program. Together, these findings support a model in which neutrophil-associated complement signals participate in inflammatory microglial remodeling during the acute phase of TBI.

Neutrophils contribute to secondary injury after TBI through inflammatory mediators, proteases, reactive oxygen species, and NET formation, and experimental studies have shown that their depletion can reduce edema, tissue loss, and glial activation (20–22). Our data extend this concept by suggesting that neutrophils may also act as signaling cells. Neutrophil recruitment coincided with the expansion of Microglia3 and Microglia4 (**Figures 1C**, **2B**), and computational analyses predicted extensive communication from neutrophils to these microglial populations (**Figures 3A–C**). Although these analyses do not establish direct cell-to-cell causality, they provide a framework for identifying molecular signals that connect peripheral immune-cell recruitment to resident glial-state remodeling.

Complement activation is an established contributor to secondary brain injury after TBI (12), with C3 serving as a central convergence point and C3a signaling through C3aR1 to promote inflammatory and neurovascular dysfunction (24–26). In our study, NicheNet prioritized C3 among candidate ligands expressed by infiltrating neutrophils, whereas *C3ar1* transcripts were enriched in inflammatory microglial states (**Figures 3D, E**). Immunofluorescence further demonstrated increased C3 signal in Ly6G-positive infiltrating neutrophils and elevated C3aR1 expression in microglia after TBI (**Figures 5A–D**), supporting the spatial association predicted by the computational analyses. These findings identify infiltrating neutrophils as a plausible local cellular source of C3 during acute TBI; however, the present study cannot distinguish the relative contributions of neutrophil-associated C3, C3 produced by other CNS cell populations, and circulating C3, including liver-derived C3. Cell type-specific genetic approaches will therefore be required to establish the causal contribution of neutrophil-associated complement signaling.

Pharmacological modulation with JR14a was associated with reduced IL-1β and TNF-α expression in microglia, preservation of Claudin-5, reduced Evans blue extravasation, and improved neurological and cognitive outcomes after TBI (**Figure 6**, **7**). These findings are consistent with previous studies showing that pharmacological complement inhibition improves neurological recovery after experimental TBI (27), whereas genetic or pharmacological disruption of C3aR1 signaling attenuates microglial activation and neurovascular dysfunction in other CNS injury and disease models (28–30). Together, these observations support C3aR1-associated signaling as a potential link between inflammatory microglial responses and neurovascular injury after TBI. From a translational perspective, targeting this pathway during the acute post-injury period may represent a potential strategy to limit neuroinflammation and preserve blood–brain barrier integrity. However, systemic JR14a administration does not establish a microglia-specific mechanism, and further studies are required to define the cell-specific contribution of C3/C3a–C3aR1 signaling, therapeutic window, pharmacological specificity, and safety before clinical translation.

The regulatory analyses further prioritized Ets2, Fosl2, and Irf7 as candidate regulators associated with the *C3ar1*-high microglial state (**Figure 4**). Previous studies have implicated these factors in inflammatory, stress-response, and metabolic programs in myeloid cells and microglia (8, 31–34), providing biological context for their computational prioritization. However, our computational analyses establish association and candidate prioritization rather than functional causality. Accordingly, the convergence of SCENIC, CellOracle, and scTenifoldKnk should be regarded as a strategy for prioritizing experimentally testable regulators rather than evidence of a defined C3aR1-dependent transcriptional pathway (**Figure 4**; **Supplementary Figure S3**). Future studies should determine whether these regulators functionally mediate C3aR1-associated microglial responses after TBI.

In summary, our findings identify a putative neutrophil-associated C3/C3a–C3aR1 communication pathway linked to inflammatory microglial remodeling after TBI. Together with the pharmacological findings, these results support further investigation of C3aR1-associated signaling as a potential therapeutic target for secondary neuroinflammation and neurovascular dysfunction after TBI.

## 4 Methods

### 4.1 Animals and traumatic brain injury model

All studies were performed in adult male C57BL/6 mice aged 8–10 weeks. Mice were purchased from the Vital River Corporation (Beijing, China). All the mice were maintained under Specific Pathogen Free (SPF) conditions and housed with no more than five animals per cage with standardized light-dark cycle conditions and provided with plenty of water and food. TBI was induced using a controlled cortical impact (CCI) model (35). Briefly, mice were anesthetized with isoflurane (2% in oxygen) and placed in a stereotaxic frame. After exposing the skull, a circular craniotomy (3.5 mm in diameter) was performed over the right parietotemporal skull region (2.5 mm posterior to the bregma and 2.5 mm lateral to the sagittal suture) using a motorized drill. CCI injury was induced using a 3-mm diameter flat-tip impactor. The impact parameters were set as follows: velocity, 5 m/s; dwell time, 200 ms; and impact depth, 2.2 mm. Following injury, the skull opening was closed, and mice were placed on a heating blanket to maintain body temperature during recovery from anesthesia.

### 4.2 Single-cell RNA sequencing and data processing

Publicly available single-cell RNA sequencing datasets of mouse traumatic brain injury were obtained from the Gene Expression Omnibus (GEO) database (GSE269748 and GSE290150) (15, 36). Samples were assigned to the Control, 1-day post-TBI (TBI1d), and 7-day post-TBI (TBI7d) groups according to the original sample metadata. The Control group included 10 samples, consisting of seven naive samples from GSE269748 and three normothermic sham replicates from GSE290150. The TBI1d group included nine samples, consisting of six samples collected 1 day after TBI from GSE269748 and three normothermic TBI replicates from GSE290150. The TBI7d group included three samples collected 7 days after TBI from GSE269748. Only normothermic samples from GSE290150 were included, whereas samples subjected to therapeutic hypothermia were excluded.

Raw count matrices and associated metadata were processed using Seurat (version 5.3.0) in R. Seurat objects were initially generated using CreateSeuratObject with min.cells = 3 and min.features = 200, such that genes detected in fewer than three cells and cells with fewer than 200 detected features were excluded at object construction. The percentage of mitochondrial transcripts was calculated using PercentageFeatureSet with the pattern “^mt-”. Quality-control metrics included the number of detected genes (nFeature_RNA), total unique molecular identifier (UMI) counts (nCount_RNA), and mitochondrial transcript percentage (percent.mt). Cells with ≤200 or ≥7,500 detected genes or ≥20% mitochondrial transcripts were excluded. Total UMI counts were examined during quality-control assessment but were not subjected to an additional fixed filtering threshold.

Following quality control, individual sample expression matrices were normalized using the Seurat NormalizeData function with the default LogNormalize method (scale factor = 10,000). Highly variable genes were identified using FindVariableFeatures with the variance-stabilizing transformation (vst) method, retaining 2,000 variable features. The variable features were scaled and subjected to principal-component analysis (PCA). To reduce technical variation among samples and between the two GEO datasets, batch correction was performed using Harmony, with individual sample identity (orig.ident) specified as the batch variable and lambda = 0.5. The first 40 Harmony dimensions were used for nearest-neighbor graph construction and graph-based clustering using FindNeighbors and FindClusters, respectively, with a clustering resolution of 0.5. Uniform Manifold Approximation and Projection (UMAP) was subsequently performed using the Harmony-corrected embedding. Following quality control and batch correction, 254,512 cells were retained for downstream analyses.

### 4.3 Differentially expressed gene and pathway enrichment

Differentially expressed genes (DEGs) between the TBI and Control groups were identified using the FindMarkers function in Seurat with min.pct = 0.25 and logfc.threshold = 0.25. Gene Ontology Biological Process (GO-BP) enrichment analysis was subsequently performed using the resulting DEGs. GO terms with adjusted *P* values < 0.05 were considered significantly enriched.

### 4.4 Gene set scoring

The gene set scores of each cell were estimated using the AddModuleScore function in Seurat, which calculated the average expression levels of each program (cluster) on single cell level, subtracted by the aggregated expression of control feature sets. All analyzed features are binned based on averaged expression, and the control features are randomly selected from each bin. Gene sets for inflammatory response, phagocytosis, and lipid metabolism are provided in **Supplementary Tables S1**-**S3**, respectively.

### 4.5 Pseudotime trajectory and BEAM analysis

Microglial state transitions were analyzed using Monocle2. Cells were ordered along pseudotime trajectories based on transcriptional similarity. Differentially expressed genes associated with pseudotime progression were identified using differentialGeneTest. Branched Expression Analysis Modeling (BEAM) was performed to identify genes exhibiting branch-dependent expression patterns during microglial state transitions.

### 4.6 Cell-cell communication analysis

The interaction intensity of information flow between cells was calculated using CellChat (version 1.6.1). To infer the cell state-specific communications, it first identified differentially expressed signaling genes across all cell groups within scRNA-seq dataset, using the Wilcoxon rank sum test with the significance level of 0.05, and then modeled ligand-receptor mediated signaling interactions using the law of mass action. The communication between neutrophils and microglia was repeatedly predicted by NicheNet (version 2.0.4). It assessed target genes and ligands with high activity ranking and inferences ligand-to-target signal transduction pathways.

### 4.7 Definition of *C3ar1*-high and *C3ar1*-low microglia

To characterize transcriptional programs associated with *C3ar1* expression, microglial cells were stratified using log-normalized *C3ar1* expression values from the RNA assay. A single global cutoff corresponding to the 75th percentile of *C3ar1* expression across all microglial cells from the Control, TBI1d, and TBI7d groups was applied. Cells with expression values above this threshold were classified as *C3ar1*-high microglia, whereas the remaining cells were classified as *C3ar1*-low microglia. Differentially expressed genes between the two groups were identified using the FindMarkers function in Seurat, followed by Gene Ontology biological process enrichment analysis.

### 4.8 Transcription factor regulatory network analysis

To identify candidate transcription factors associated with *C3ar1*-high microglia, complementary computational approaches were applied.

SCENIC analysis was performed to infer transcription factor–target gene regulatory networks and calculate regulon activity using AUCell. Regulon specificity scores were used to identify transcription factors enriched in *C3ar1*-high microglial populations.

CellOracle was applied to construct gene regulatory networks and perform in silico transcription factor perturbation analysis. Candidate transcription factors were virtually perturbed, and the resulting perturbation-induced cell-state transition vectors were compared with the reference pseudotime/state-transition vector field. The perturbation score was calculated as the inner product between the perturbation-induced vector and the reference transition vector, thereby incorporating both vector direction and magnitude. Positive perturbation scores indicate alignment with the inferred state-transition direction, whereas negative scores indicate opposition to that direction. These scores represent computational predictions of changes in cell-state dynamics rather than statistical significance or directly measured gene-expression changes. CellOracle analysis was performed following the official tutorial with default parameters (https://morris-lab.github.io/CellOracle.documentation/).

### 4.9 scTenifoldKnk virtual knockout and downstream functional analysis

To predict the potential regulatory consequences of candidate transcription factor perturbations in *C3ar1*-high microglia, we performed in silico knockout analysis using the scTenifoldKnk framework. Prior to network construction, low-quality genes and genes associated with technical variation, including ribosomal and mitochondrial genes, were filtered from the expression matrix. The filtered count matrix was subsequently used for construction of the baseline gene regulatory network. Virtual knockout analysis was performed by individually perturbing candidate transcription factors identified from SCENIC analysis, including Ets2, Fosl2, and Irf7. For each transcription factor, scTenifoldKnk reconstructed gene regulatory networks before and after in silico perturbation and calculated differential regulatory effects between the original and perturbed networks. Genes showing strong predicted perturbation effects were identified using a prespecified Z-score threshold (Z > 1.96), following a previously published scTenifoldKnk-based analytical framework (37, 38). The perturbation Z-score represents a Box–Cox-transformed and standardized differential-regulation distance and was interpreted as a measure of relative perturbation magnitude rather than as an independent P value. To investigate the biological functions associated with predicted transcription factor perturbations, downstream enrichment analyses were performed using the perturbed gene sets. GO-BP enrichment analysis was conducted using clusterProfiler with Benjamini–Hochberg correction for multiple testing. Biological pathways associated with significantly perturbed genes were identified based on adjusted *P* values.

### 4.10 Immunofluorescence staining

Following euthanasia with isoflurane, mice were perfused with PBS via cardiac puncture to remove intravascular blood. Brains were collected, embedded in OCT cryomedium (cat. no. 3801480, Leica), snap-frozen in liquid nitrogen and cryosectioned at 12 µm. For immunofluorescence staining, sections were washed with PBS and blocked for 1 h at room temperature in 5% BSA containing 0.3% Triton X-100. Sections were then incubated overnight at 4 °C with primary antibodies against CD31 (cat. no. AF3628, R&D), Claudin-5 (cat. no. 34-1600, Invitrogen), Ly6G (cat. no. ab238132, Abcam), IBA1 (cat. no. ab178846, Abcam), C3aR1 (cat. no. AP58066, Abcepta) and C3 (cat. no. 79981031, Invitrogen). After washing with PBS, sections were incubated with Alexa Fluor-conjugated secondary antibodies and counterstained with 4′,6-diamidino-2-phenylindole (DAPI; Sigma-Aldrich, St. Louis, MO, USA) to visualize nuclei.

### 4.11 Flow cytometry analysis

As previously described (39), flow cytometry was used to detect microglia in mice brain. Mouse brain was harvested after perfusion with cold PBS, minced and incubated with collagenase IV at 37 °C for 30 minutes. After PBS washing, myelin debris was removed by centrifugation in 30% Percoll (Cytiva), and the cell pellet was washed with PBS and resuspended in 1% BSA and incubated with Fixable Viability Stain 780 (BD Biosciences), PE/Cyanine7 anti-mouse CD45 Antibody (Biolegend), FITC anti-mouse/human CD11b Antibody (Biolegend), BV650 rat anti-mouse C3aR antibody (BD Biosciences) for 30 minutes at 4°C to identify CD45^int^CD11b^+^ microglia and quantify C3aR1 and intracellular cytokine expression. Intracellular flow cytometry (ICFC) was performed to detect the expression of PE anti-mouse IL-1β antibody (eBioscience) and Pacific blue anti-mouse TNF-α antibody (Biolegend). Briefly, cells were fixed in fixation buffer for 20 minutes after surface marker staining, washed with permeabilization buffer, stained with antibodies against intracellular molecules for 45 minutes, washed again and resuspended in flow cytometry buffer. Flow cytometry data were acquired using (LSR II, BD Biosciences, San Jose, CA) and analyzed using FlowJo software.

### 4.12 JR14a treatment

Beginning within 1 h after TBI induction, mice received JR14a (TargetMol, catalog no. T9316) at 2 mg/kg via the tail vein to pharmacologically modulate C3aR1-associated signaling. For the acute experimental cohort used for inflammatory, blood–brain barrier, and histological assessments (**Figure 6**), mice received a single dose of JR14a within 1 h after TBI and were euthanized 24 h after injury. For the behavioral cohort (**Figure 7**), the first dose of JR14a was administered within 1 h after TBI, followed by once-daily administration through post-injury day 7. No further JR14a was administered after day 7. Mice were randomly assigned to the experimental groups, and treatment administration and outcome assessments were performed by investigators blinded to group allocation. Subsequent experimental assessments were conducted at the indicated time points after treatment.

### 4.13 Evans blue permeability assay

As previously described (40), blood–brain barrier permeability was assessed using Evans blue extravasation. On day 1 after TBI, mice received an intravenous injection of 2% EB solution at a dose of 50 μL per 10 g body weight. For assessment of blood–brain barrier permeability, mice were euthanized 2 h after EB administration and perfused with ice-cold phosphate-buffered saline (PBS) before brain tissue collection. The harvested tissues were homogenized in 1 mL formamide and incubated at 70 °C for 48 h to extract the dye. Tissue homogenates were then centrifuged at 15,000 × g for 1 min, and the supernatants were transferred to 96-well plates. Evans blue content was quantified by measuring absorbance at 620 nm using a microplate reader.

### 4.14 Morris water maze test

Eight or nine mice per group were subjected to the Morris water maze (MWM) test. Acquisition trials began on day 14 after TBI and were conducted for five consecutive days (days 14–18). Mice were tested in a randomized order and released into the water facing the wall of the pool. Each mouse was allowed 60 s to locate and climb onto the submerged platform. Escape latency, total swimming distance and swimming speed were recorded using a computerized video-tracking system. Mice that failed to locate the platform within 60 s were guided to it and allowed to remain there for 30 s. Their escape latency was recorded as 60 s.

The spatial probe trial was conducted on day 19, one day after the final acquisition session. The platform was removed, and each mouse was allowed to swim freely for 60 s. The time spent in the target quadrant and the number of crossings over the former platform location were recorded. Swimming speed was also measured to control for potential differences in motor function. Data from the acquisition and probe trials were recorded and analyzed using EthoVision XT 16.

### 4.15 Neurological score assessment

Neurological function was evaluated using the modified neurological severity score (mNSS) on days 1, 3 and 7 after TBI. The mNSS assesses motor, sensory, reflex and balance functions. One point was assigned for each task that the mouse failed to perform or each tested reflex that was absent. The total score ranged from 0 to 18, with higher scores indicating greater neurological impairment. A score of 0 indicated normal neurological function, whereas scores of 1–6, 7–12 and 13–18 indicated mild, moderate and severe impairment, respectively. Assessments were performed by two investigators who were blinded to the group assignments.

### 4.16 Rotarod test

Motor coordination and balance were assessed using an accelerating rotarod apparatus (RWD Life Science, Shenzhen, China). Before TBI, mice underwent an initial 30 s acclimation period on a stationary rod, followed by three consecutive days of training. Each training day comprised three trials separated by 30 min intervals. During each trial, mice remained on the rod at 4 rpm for 1 min, after which the rotational speed increased from 4 to 40 rpm over 5 min.

Rotarod performance was assessed on days 1, 3 and 7 after TBI. During each test, the rotational speed increased from 4 to 40 rpm over 5 min, and the latency to fall was recorded. Each mouse completed three trials per day, with a 30-min interval between trials. The mean latency to fall across the three trials was used for statistical analysis. All tests were performed by two investigators who were blinded to the animal group assignments.

### 4.17 Statistical analysis

Statistical analyses were performed using GraphPad Prism and R software.

Data are presented as mean ± SEM. Comparisons between two groups were performed using an unpaired/paired Student’s t-test or Mann–Whitney U test when appropriate. Multiple group comparisons were performed using one-way ANOVA followed by Tukey’s multiple comparisons test. For experiments involving both treatment group and time as factors, two-way ANOVA followed by appropriate *post hoc* multiple-comparisons tests was used. For single-cell transcriptomic analyses, statistical methods specific to each computational approach were applied as described above. Adjusted P values were used for multiple testing correction where appropriate. A value of P < 0.05 was considered statistically significant.

## Data Availability

The single-cell RNA-sequencing datasets analyzed in this study are publicly available in the Gene Expression Omnibus (GEO) database under accession numbers GSE269748 and GSE290150. The additional data generated during this study are available from the corresponding authors upon reasonable request.
