# Supplementary material for: Neutrophil–microglia communication through the C3–C3aR1 axis contributes to secondary neuroinflammation after traumatic brain injury
Source: Front Immunol. 2026 Oct 23;17:1979527. doi: 10.3389/fimmu.2026.1979527 (PMC13739808; doi:10.3389/fimmu.2026.1979527)
Supplement: Supplementary file 1 [file DataSheet1.pdf]

frontiers

The International Society for the Advancement of Cytometry (ISAC) has highlighted the importance of including comprehensive methodological information to ensure data reproducibility and reliability. In line with this, Frontiers in Immunology now requires authors to submit a checklist for manuscripts that involve flow or mass cytometry. This checklist helps standardize the reporting process, improving the quality and transparency of published data. By doing so, we support scientific progress, making it easier for other researchers to replicate and validate experiments.

This form should be submitted with any manuscripts using flow or mass cytometry.

**Sample/specimen/material description**

☐ Total blood

☐ PBMCs

☑ Organ digests

Other_______________________________________

Did the samples suffer any treatment before or after incubation with the antibodies?

☑ Drug ________________________________________

☐ Cell permeabilization _________________________

☐ Dye __________________________________________

☐ Propidium iodine

☐ Not applicable

Other _________________________________________

**Instrument and antibodies**

Name of the Cytometer LSR II, Becton Dickinson

| Antibodies and targets | Fluorochrome/ Metal | Catalog number/Company |
|---|---|---|
| e.g. anti-CD4 | FITC | Cat. XXX/ XXX ltd. |
| anti - CD45 | PE/Cyanine7 | Cat. 157614/Biolegend |

frontiers

| | | |
|---|---|---|
| anti - CD11b | FITC | Cat. 101205/Biolegend |
| anti - C3aR1 | BV650 | Cat. 753360/BD Biosciences |
| anti - IL-1 | PE | Cat. 12-7114-82/eBioscience |
| anti - TNF | Pacific blue | Cat. 506318/Biolegend |
| anti - Fixable Viability Stain 780 | APC-CY7 | Cat. 565388/BD Biosciences |
| | | |
| | | |
| | | |
| | | |
| | | |
| | | |
| | | |
| | | |
| | | |
| | | |
| | | |
| | | |
| | | |
| | | |
| | | |
| | | |
| | | |
| | | |
| | | |
| | | |
| | | |
| | | |
| | | |
| | | |

| | | |
|---|---|---|
| | | |
| | | |
| | | |
| | | |
| | | |
| | | |
| | | |
| | | |
| | | |
| | | |
| | | |
| | | |
| | | |
| | | |
| | | |
| | | |
| | | |
| | | |
| | | |
| | | |

**Data analyses**

1. Name of the software FlowJo________________________________
2. Reference gating strategy in the manuscript or supplementary material

Gating strategy in (eg Figure X) Figure S4______________________________
