# Supplementary material for: Neutrophil–microglia communication through the C3–C3aR1 axis contributes to secondary neuroinflammation after traumatic brain injury
Source: Front Immunol. 2026 Oct 23;17:1979527. doi: 10.3389/fimmu.2026.1979527 (PMC13739808; doi:10.3389/fimmu.2026.1979527)
Supplement: Supplementary file 2 [file Supplementaryfile1.pdf]

SUPPLEMENTAL RESULTS

SUPPLEMENTAL TABLE

Table S1: The gene sets of cells for inflammation response.

Acvr1	Bdkrb2	Crp	F2	Fut7	Il31ra	Mrgpra3	Pla2g2d	Saa2	Stat3
Adam8	Btk	Ctnnbip1	F3	Gata3	Il4	Nlrp3	Plscr1	Saa3	Stat5b
Adcyap1	C2cd4a	Cxcr2	F8	Gstp1	Il6	Nlrp6	Pparg	Saa4	Tac1
Adora1	C2cd4b	Dnase1	Fcer1a	Hp	Ins1	Npff	Prcp	Scn11a	Tfrc
Ahsg	C3	Dnase1l3	Fcer1g	Icam1	Ins2	Npy	Ptger3	Selenos	Tnf
Alox5ap	Casp6	Ednrb	Fcgr1	Ighg1	Itih4	Npy5r	Ptges	Serpina1a	Tnfrsf11a
Ano6	Ccl5	Eif2ak1	Fcgr2b	Ighg2b	Klk1b1	Nupr1	Ptgs2	Serpina1b	Tnfsf11
Aoc3	Ccr5	Elane	Fcgr3	Ighg3	Lbp	Orm1	Reg3a	Serpina3n	Tnfsf4
Apcs	Ccr7	Ephb6	Fcgr4	Il1a	Lcn2	Orm2	Reg3b	Serpinb9	Trpv1
Ash1l	Cd163	Epo	Ffar2	Il1b	Lipa	Orm3	Reg3g	Serpinf2	Vnn1
Ass1	Cd6	Ext1	Ffar3	Il1rn	Mptx1	Park7	Rhbdd3	Sigirr	Vwf
B4galt1	Cnr1	F12	Fn1	Il20rb	Mptx2	Pik3cg	Saa1	Spn	Zp3

Table S2: The gene sets of cells for phagocytosis.

Abca1	Atg3	Cd93	Dysf	Ifng	Lyn	Pear1	Rab11fip2	Slamf1	Tlr4
Abca7	Atg5	Cdc42	Eif2ak1	Il15	Lyst	Pecam1	Rab14	Slc11a1	Tm9sf4
Abl1	Axl	Cdc42se1	Elane	Il15ra	Marco	Pik3ca	Rab20	Slc48a1	Tmem175
Adgrb1	Bcr	Cdc42se2	Elmo1	Il2rb	Mbl2	Pikfyve	Rab27a	Snx3	Trem2
Adipoq	Becn1	Cebpe	Elmo2	Il2rg	Megf10	Pip4p2	Rab31	Sod1	Trex1
Adora1	Bin2	Cfp	Elmo3	Irf8	Mertk	Pip5k1a	Rab34	Spaca3	Tub
Adora2a	Bltp1	Clcn2	F2rl1	Itga2	Mesd	Pip5k1c	Rab39	Spg11	Tulp1
Ager	Btk	Clcn3	Fcer1g	Itgal	Mfge8	Pla2g5	Rab5a	Sphk1	Tusc2
Ahsg	Pycard	Clec7a	Fcer2a	Itgam	Msr1	Pla2g6	Rab7	Spon2	Tyro3
Aif1	C2	Cln3	Fcgr1	Itgav	Mst1r	Plcg2	Rab7b	Src	Tyrobp
Alox15	C3	Cnn2	Fcgr2b	Itgb1	Myd88	Pld2	Rac1	Srpx	Unc13d
Ano6	C4b	Colec10	Fcnb	Itgb2	Myh9	Pld4	Rack1	Stap1	Vamp7
Anxa1	C4bp	Colec11	Fgr	Itgb3	Myo18a	Plpp4	Rap1a	Stat3	Vav1
Anxa11	Calr	Colec12	Fpr2	Jmjd6	Myo1g	Plscr1	Rap1gap	Syk	Vav2
Anxa3	Camk1d	Coro1a	Fyn	Lbp	Myo7a	Prkaca	Rara	Syt11	Vav3
Apoa1	Ccl2	Coro1c	Gas6	Ldlr	Ncf2	Prkcd	Rubcn	Syt7	Vipas39
Apoa2	Ccr2	Crp	Gata2	Lep	Ncf4	Prkce	Scarb1	Tafa4	Vps33b
Appl1	Cd14	Cryba1	Gsn	Lepr	Nckap1l	Prkcg	Sftpa1	Tgfb1	Xkr4
Appl2	Cd300a	Csk	Gulp1	Letmd1	Nos2	Prtn3	Sftpd	Tgm2	Xkr6
Arap1	Cd300lf	Cyba	Havcr1	Limk1	Nr1h3	Ptk2	Sh3bp1	Thbs1	Xkr7

Arhgap12	Cd302	Dnm2	Hck	Lman2	P2rx7	Ptprc	Sirpa	Ticam2	Xkr8
Arhgap25	Cd36	Dock1	Hmgb1	Lrp1	P2ry6	Ptprj	Sirpb1a	Timd4	Yes1
Arl8b	Cd47	Dock2	Icam5	Lyar	Pak1	Ptx3	Sirpb1b	Tlr2	

Table S3: The gene sets of cells for lipid metabolism.

Aadac	Akt1	Ccdc3	Daglb	Gpld1	Ldlr	Nr1h3	Ppard	Scp2	Tafazzin
Abcd1	Akt2	Ccn1	Dgat2	Hsd17b13	Ldlrap1	Nr1h4	Pparg	Sct	Tnf
Abcd2	Angptl3	Cd74	Disp3	Htr2a	Lpgat1	Nr5a2	Ppargc1a	Sirt2	Tnfrsf1a
Abcg1	Apoa1	Ces1d	Eef1a2	Htr2b	Mapk1	Nsmaf	Prkaa1	Sirt3	Twist1
Abcg4	Apoa2	Cga	Elovl5	Htr2c	Mbtps2	Ogt	Prkaca	Sirt4	Wnt4
Abhd5	Apoa4	Chp1	Enpp7	Ifng	Mfsd2a	Paqr3	Prkcd	Slc27a1	Xbp1
Abhd6	Apoa5	Clstn3	Fabp3	Il1b	Mid1ip1	Pck1	Prkce	Slc45a3	Zbtb20
Acsl3	Apoc2	Cnep1r1	Fgf1	Ins2	Mlst8	Pla2g3	Ptgs2	Sorbs1	Zfp750
Adgrf5	Apoe	Cpt1a	Fshb	Irs1	Mlxipl	Pla2g6	Qki	Spata18	
Adipoq	Apoh	Creb1	Fut1	Irs2	Mlycd	Plaa	Rab38	Sphk2	
Adm	Avp	Crebl2	Gdf15	Kat2b	Mtln	Plin5	Rdh10	Srebf1	
Adora1	Avpr1a	Ctdnep1	Ghsr	Kat5	Mtor	Pnpla2	Rptor	Srebf2	
Agt	Bmp6	Cyp7a1	Gnai1	Klhl25	Nr1d1	Por	Scap	Star	
Agtr1a	Capn2	Dab2	Gpihbp1	Kpnb1	Nr1h2	Ppara	Scarb1	Stard4	

SUPPLEMENTAL FIGURES AND LEGENDS

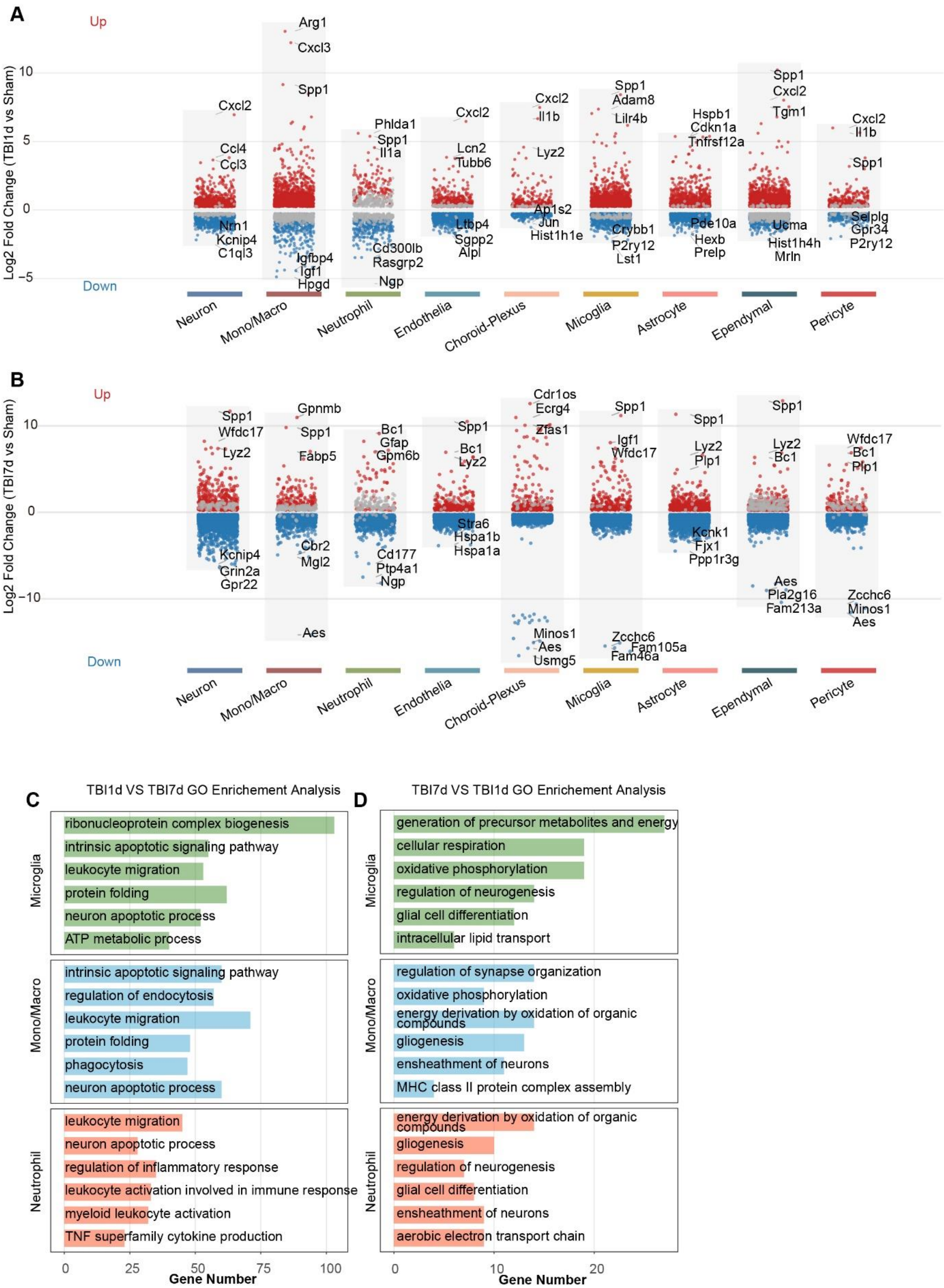

Figure S1. Global transcriptional alterations and dynamic functional shifts across brain cell populations following TBI. (A–B) Plots displaying global differential gene expression

across all identified cell types at 1 day (TBI1 vs. control) (A) and 7 days (TBI7 vs. control) (B) post-injury. Each dot represents an individual gene. Red and blue dots indicate significantly upregulated and downregulated genes, respectively. **(C–D)** GO biological process enrichment analysis of differentially expressed genes between TBI1 and TBI7. Bar plots show enriched biological processes in microglia, monocyte/macrophage, and neutrophil populations, with gene numbers indicated on the x-axis.

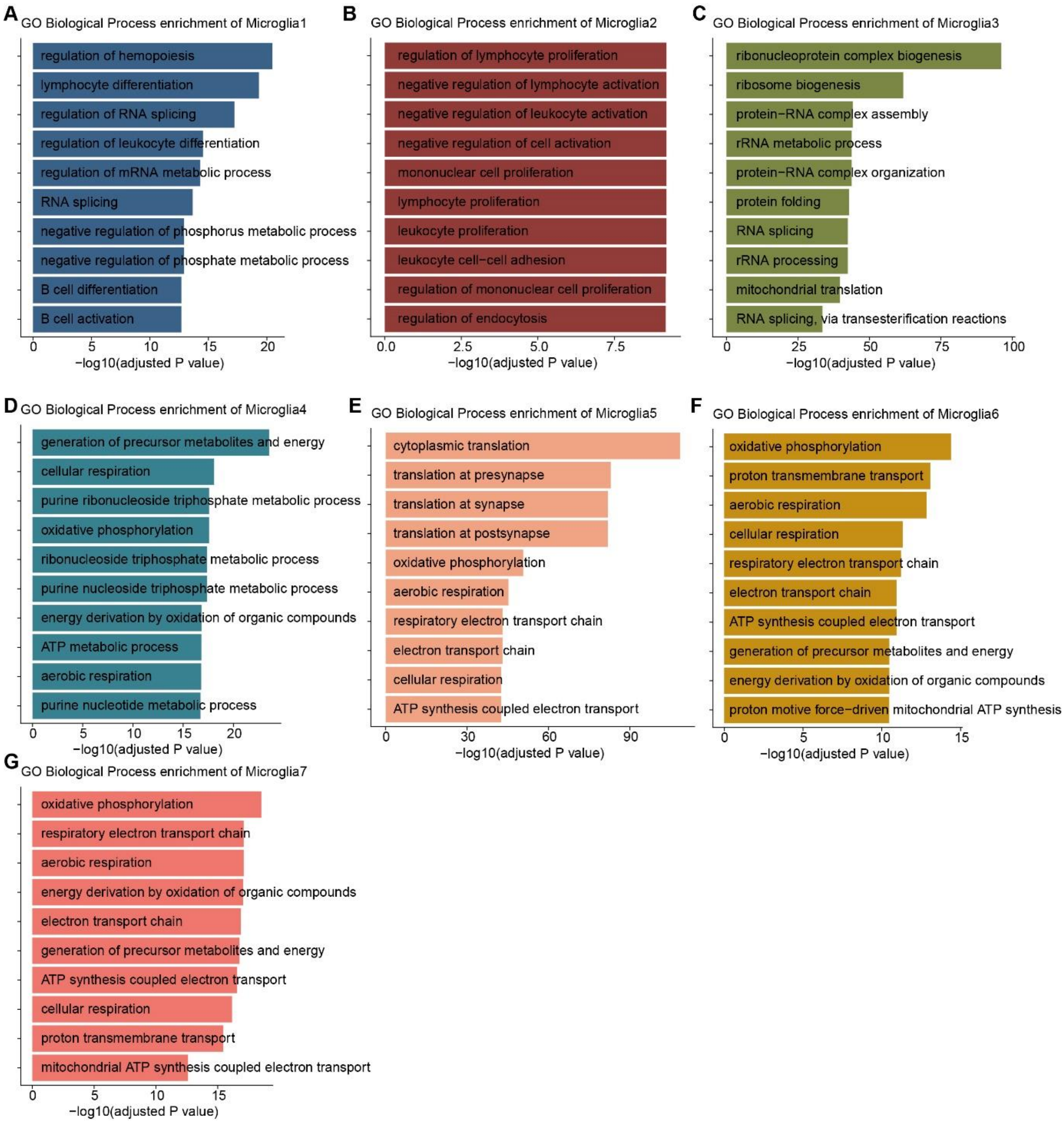

Figure S2: Functional annotation of microglial subclusters based on GO enrichment analysis. (A-G) GO biological process enrichment analysis was performed using cluster-specific differentially expressed genes from seven microglial subclusters. Bar plots show representative enriched biological processes ranked by adjusted P value.

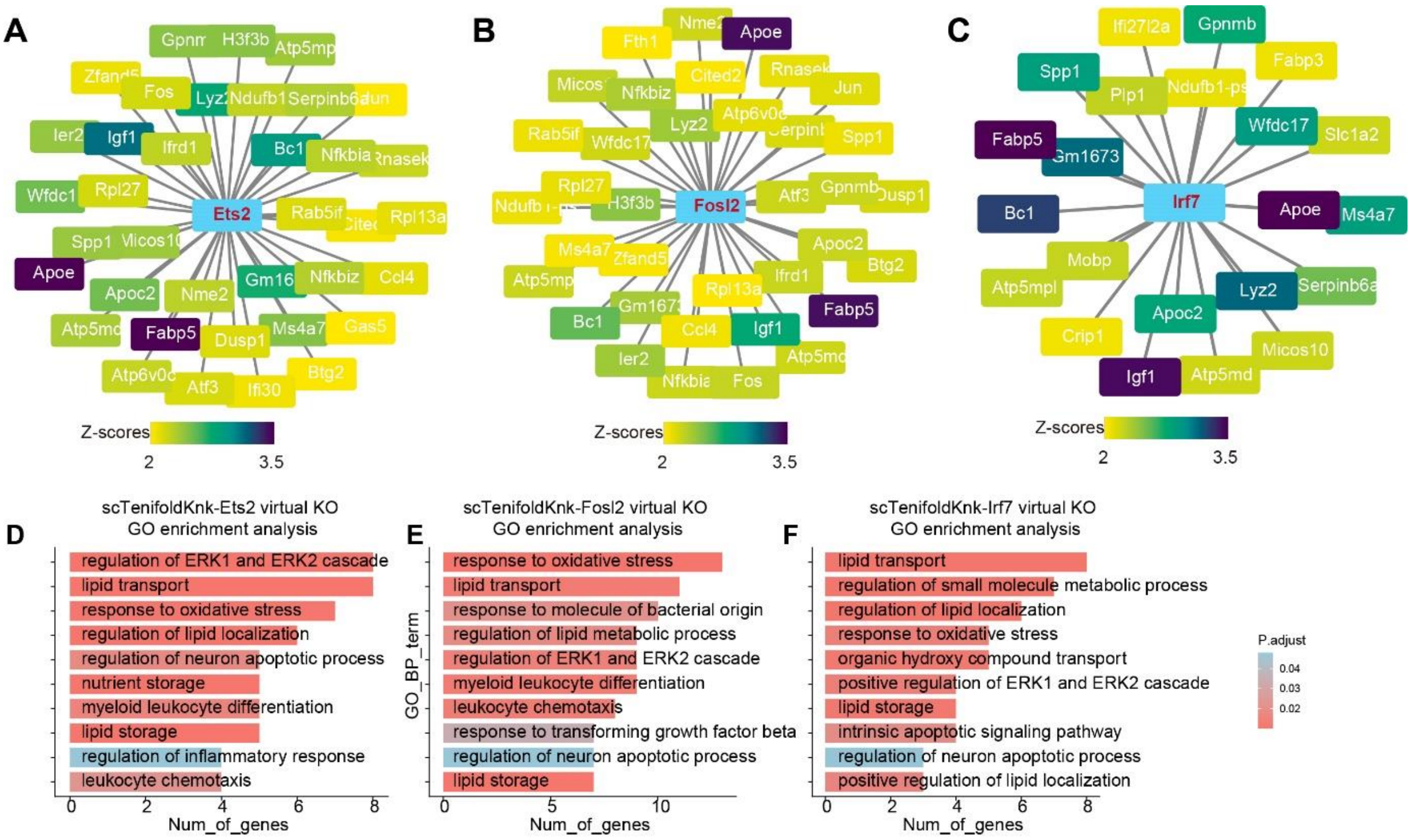

Figure S3: scTenifoldKnk virtual knockout predicts perturbation-sensitive gene networks associated with candidate transcription factors in *C3ar1-high* microglia. (A–C) Network visualization of genes predicted to be most strongly affected following the virtual knockout of *Ets2* (A), *Fosl2* (B), and *Irf7* (C) in *C3ar1*-high microglia. Node colors represent scTenifoldKnk-derived perturbation Z-scores, with higher Z-scores indicating greater predicted network-level changes following virtual knockout. **(D-F)** GO enrichment analysis of differentially perturbed genes predicted by scTenifoldKnk following Ets2 (D), Fosl2 (E) and Irf7 (F) virtual knockout.

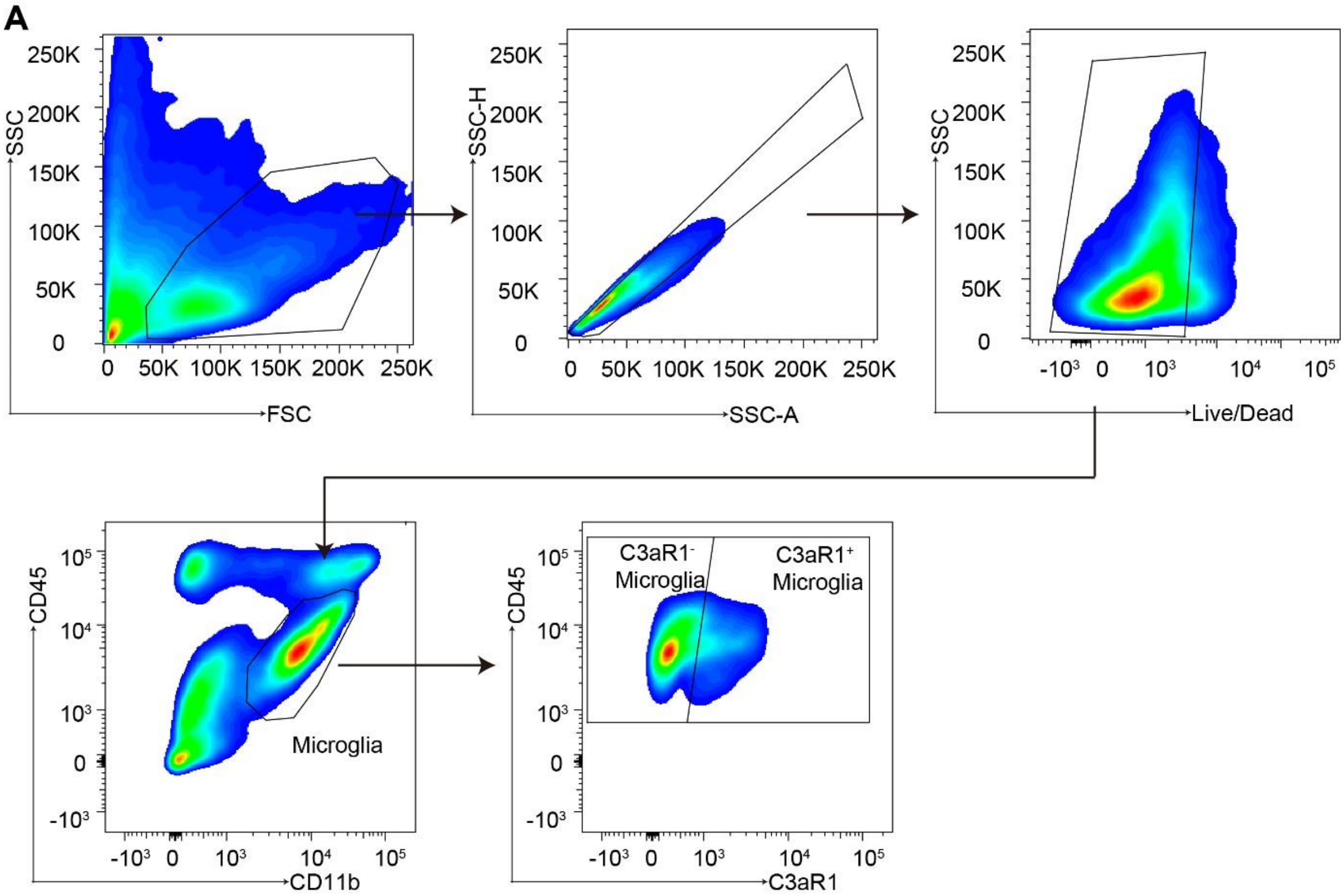

Figure S4: Representative flow-cytometry gating strategy for identification of C3aR1⁺ microglia. (A) Representative gating strategy showing sequential identification of the microglial population and assessment of C3aR1 expression. Cells were first gated based on forward scatter (FSC) and side scatter (SSC), followed by exclusion of doublets using SSC-A versus SSC-H and selection of viable cells using Live/Dead staining. Microglia were subsequently identified as $CD45^{int}CD11b^{+}$ cells, and C3aR1 expression was assessed within the gated microglial population. $C3aR1^{-}$ and $C3aR1^{+}$ microglia were then distinguished according to C3aR1 fluorescence intensity.

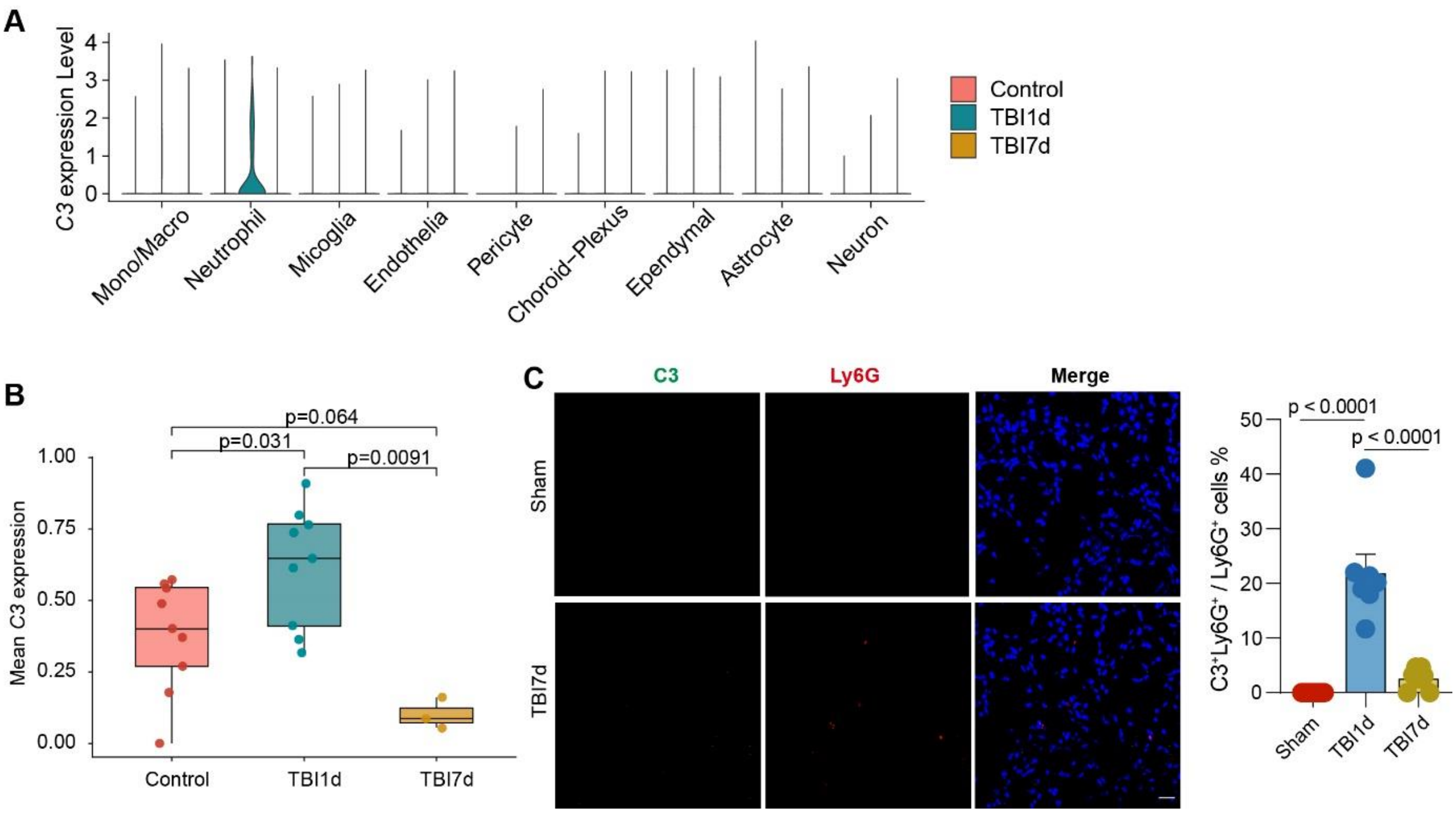

Figure S5: Temporal dynamics of neutrophil-associated C3 expression after TBI. (A) Violin plots showing *C3* expression across major brain cell populations in Control, TBI1d, and TBI7d groups based on single-cell RNA-sequencing data. **(B)** Mean *C3* expression in neutrophils among Control, TBI1d, and TBI7d groups. Mean *C3* expression was calculated separately for neutrophils from each sample. **(C)** Representative immunofluorescence images showing C3 (green), Ly6G (red), and nuclei (DAPI, blue) in brain tissue from Sham and TBI7d mice. The TBI1d quantitative data are the same dataset presented in Figure 5D and are included here to allow direct comparison of the temporal changes in neutrophil-associated C3 expression. (bar = 20 μm, n = 6-7/group, one-way ANOVA test). Data are presented as mean ± SEM.
